# Abnormal Habituation of the Auditory Event-Related Potential P2 Component in Patients With Schizophrenia

**DOI:** 10.3389/fpsyt.2021.630406

**Published:** 2021-03-18

**Authors:** Prune Mazer, Inês Macedo, Tiago O. Paiva, Fernando Ferreira-Santos, Rita Pasion, Fernando Barbosa, Pedro Almeida, Celeste Silveira, Cassilda Cunha-Reis, João Marques-Teixeira

**Affiliations:** ^1^Laboratory of Neuropsychophysiology, Faculty of Psychology and Education Sciences of the University of Porto, Porto, Portugal; ^2^School of Health, Polytechnic Institute of Porto, Porto, Portugal; ^3^Faculty of Law, School of Criminology and Interdisciplinary Research Center on Crime, Justice and Security, University of Porto, Porto, Portugal; ^4^Faculty of Medicine, University of Porto, Porto, Portugal; ^5^Psychiatry Department, Hospital S. João, Porto, Portugal

**Keywords:** schizophrenia, auditory, EEG, event-related potentials, habituation, N1, P2

## Abstract

Auditory event-related potentials (ERP) may serve as diagnostic tools for schizophrenia and inform on the susceptibility for this condition. Particularly, the examination of N1 and P2 components of the auditory ERP may shed light on the impairments of information processing streams in schizophrenia. However, the habituation properties (i.e., decreasing amplitude with the repeated presentation of an auditory stimulus) of these components remain poorly studied compared to other auditory ERPs. Therefore, the current study used a roving paradigm to assess the modulation and habituation of N1 and P2 to simple (pure tones) and complex sounds (human voices and bird songs) in 26 first-episode patients with schizophrenia and 27 healthy participants. To explore the habituation properties of these ERPs, we measured the decrease in amplitude over a train of seven repetitions of the same stimulus (either bird songs or human voices). We observed that, for human voices, N1 and P2 amplitudes decreased linearly from stimulus 1–7, in both groups. Regarding bird songs, only the P2 component showed a decreased amplitude with stimulus presentation, exclusively in the control group. This suggests that patients did not show a fading of neural responses to repeated bird songs, reflecting abnormal habituation to this stimulus. This could reflect the inability to inhibit irrelevant or redundant information at later stages of auditory processing. In turn schizophrenia patients appear to have a preserved auditory processing of human voices.

## Introduction

The orienting response to novel/rare stimuli is considered a fundamental reaction in living organisms. However, when confronted with the repetition of stimuli in stable and non-threatening conditions, neural responses are expected to fade with repetition, demonstrating that humans tend to adapt to the surrounding environment (1). Auditory event-related potentials (ERP) and early components such as the N1 and P2, allow tracking the time course of neural activity related to auditory processing (2) and respective habituation (i.e., component amplitudes decrease with successive repetitions) and sensory gating processes (i.e., inhibition of the processing of repeated information).

N1 is a negative component that peaks around 100 ms after the onset of an auditory stimulus and is followed by the P2, a positive component occurring approximately after 200 ms (3). N1 and P2 are sensitive to the exogenous physical properties of the stimuli (4), although they also encompass endogenous characteristics (2, 3). At the exogenous level, early auditory categorical differences have been reported for distinct categories of sounds (e.g., voices and bird songs) such that results suggest a rapid brain discrimination of human voices (5–8). At the endogenous level, N1 also seems to reflect a selective-orienting attention response toward novel/rare stimuli (9, 10), while P2 probably represents a subsequent stage of stimulus recognition toward specific stimuli (e.g., human voices) independently of attentional demands (5–7, 11).

Regarding habituation processes, the N1-like orienting neural responses are likely to decrease in magnitude with the repeated presentation of an auditory stimulus (1, 9). Similarly, P2 also shows habituation and sensory gating properties, especially for the consecutive stimuli repetition with shorter and constant inter-stimulus intervals (1, 4, 12). Nevertheless, the mechanisms underlying ERP adaptation are complex (13) and further investigation is still required in order to unveil their potential clinical applications.

Several studies have focused indeed on the potential of auditory ERP components and habituation properties (e.g., P50 and P300) as diagnostic tools for schizophrenia (14, 15). N1 and P2 can be particularly useful to study the mechanisms underlying the pathophysiology of schizophrenia since they are especially sensitive to early cortical auditory processing (that appears to be affected by this disorder) and seem to arise from temporal lobe generators, which are also affected in schizophrenia (9, 16). More specifically, the ability to filter out irrelevant or repetitive information—as assessed through sensory gating and habituation processes—maps early pre-attentive and later-stages of information processing, respectively (17). Research consistently reports reduced N1 amplitudes in first-episode, as well as chronic schizophrenic patients and first-degree relatives using both simple (18–21) and complex stimuli (22, 23). Conversely, P2 is often examined in the context of the N1/P2 complex, and thus it has been less studied independently. Studies that have examined both components show either smaller P2 amplitudes (19) or no differences in first-episode schizophrenia (using simple stimuli) compared to healthy controls (21), despite systematic reductions in N1. A meta-analysis by Ferreira-Santos et al. (24) reviewed 20 studies on P2 in schizophrenia and showed that patients exhibited smaller amplitudes to standard stimuli and larger amplitudes for target stimuli when compared to controls in oddball tasks. In addition, even if fewer studies have assessed habituation processes for N1 and P2, there is evidence of less attenuation in schizophrenia (25, 26), although a meta-analysis by Rosburg (27) found that patients with schizophrenia display reduced N1 gating due to the initial/novel stimulus—not to effects of repetition/habituation. Such abnormalities extend to the processing of voice stimuli with N1 being smaller to pre-recorded voices (23, 28) and P2 exhibiting larger amplitudes for voices with affective content (29) and no differences in amplitude for neutral content (30) in Schizophrenia.

Studying voice processing in schizophrenia is particularly important since auditory verbal hallucinations and abnormal social cognition represent well-recognized symptoms of this pathology. Also, the study of pre-attentive and later-stages of information processing could help us understand which stages of the auditory cortical information processing are impaired (and which are intact) in schizophrenia, as well as if the type of stimuli (i.e., complex, simple, or social) interferes with this process. Since neurobiological impairments precede the beginning of a full clinical syndrome, it is of extreme relevance to study the N1 and P2 in pre-psychotic and early-psychotic states of schizophrenia (31) to explore deficits underlying genetic susceptibility for schizophrenia (9, 20). For this purpose, the current study assessed the habituation of auditory ERP components in first-episode schizophrenia such that amplitude attenuations at N1 and P2 time-windows were measured in a roving paradigm including both complex sounds (human voices and bird songs) and a target/rare pure sinusoidal tone. In healthy participants, the reviewed literature indicates that (a) N1 increases for rare stimuli vs. frequent stimuli (9, 10), (b) P2 is particularly responsive to human voices vs. bird sounds (5), and (c) both components decrease as a function of repetition/habituation (1, 4, 12, 32). Inversely, we hypothesized that patients with schizophrenia would show: (H1) a diminished N1 amplitude for target stimuli—pure tones (9); (H2) a diminished P2 to human voices, considering P2 specificity to voices (5) and deficits in voice processing in schizophrenia (33); and (H3) reduced habituation of N1 and P2 when compared to controls (25), considering deficits reported in sensory gating in early ERP components in schizophrenia (34).

## Method

### Participants

Twenty-seven first-episode patients diagnosed with schizophrenia were originally recruited, but one was excluded due to excessive noise in EEG recordings. Thus, the clinical group included 26 participants (8 female; 2 left-handed), with a mean age of 27.88 years (*SD* = 11.94) and a mean education of 11.73 years (*SD* = 2.69). All patients were medicated with second generation antipsychotics corresponding to, on average, 12.63 mg of olanzapine equivalents (*SD* = 5.01). Mean illness duration was 15.11 months (*SD* = 8.01). The control group included 27 healthy individuals (seven female; two left-handed), with a mean age of 27.56 years (*SD* = 7.79) and a mean education of 13.67 years (*SD* = 2.26). All participants reported being free from head injuries, other neurological disorders, learning disabilities, or substance abuse, and all disclosed having normal hearing. There were statistically significant differences between groups for education (*p* =0.006) but not for age (*p* = 0.897). Although education varied between groups, all participants in the patient group had completed at least compulsory school (9 years), except for one who completed 8 years. All participants signed the informed consent before the beginning of the experiment.

### Stimuli and Procedures

The selection of auditory stimulus for the task was based on a preliminary validation study (for more details see section Validation study of the auditory stimuli in the Supplementary Material). Using a modified version of the roving standard frequency paradigm (35), auditory stimuli (i.e., bird songs, voices, and the pure tone) were delivered via headphones and E-Prime 2.0 (Psychology Software Tools, Pittsburgh, PA) with an intensity of 90 dB SPL.

**Figure 1:**
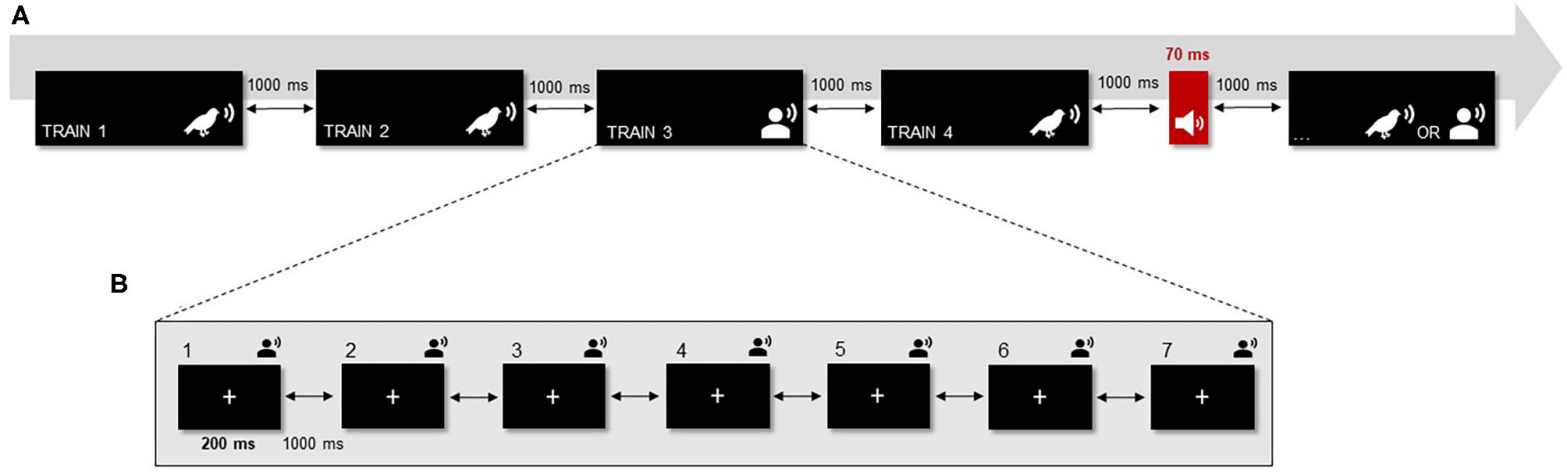
Modified version of the roving standard frequency paradigm (Baldeweg et al., 2004). **(A)** There were 16 complex auditory stimuli divided into two categories: eight bird songs and eight human voices (non-speech voices). Each complex sound was presented in trains composed of seven repetitions of the same sound (ISI = 1,000 ms). Each train was randomly repeated five times during the protocol, resulting in a total of 80 blocks. Additionally, a target tone (i.e., a pure tone with duration of 70 ms) was randomly presented 40 times in between the trains of complex sounds. **(B)** Within the trains, each sound was presented for 200 ms (ISI = 1,000 ms) while a black screen with a fixation point was displayed.

The protocol included 16 complex auditory stimuli (i.e., eight bird songs and eight non-speech human voices) with a duration of 200 ms each (10 ms rise and fall times). The complex sounds were presented in fixed trains composed of seven repetitions of the same sound with an inter-stimulus interval of 1,000 ms. Each train was randomly repeated 5 times during the protocol, resulting in a total of 80 blocks. The target tone was randomly presented 40 times during the entire protocol (i.e., probability of ~7%) and only appeared between trains (Figure 1). This target consisted of a 1,000 Hz pure tone with 70 ms duration (10 ms rise and fall times). During the task, a black screen with a fixation point was presented. Behavioral data was collected on the target tone with the main purpose of maintaining participants' attention and avoiding sleepiness and fatigue (which could impair EEG data quality due to alpha activity). Thus, participants were asked to pay attention and rapidly identify when the target tone was presented by pressing a button (i.e., in a SRBOX display; E-Prime).

### EEG Data Collection and Pre-processing

EEG data were recorded with a digitizing rate of 500 Hz from 128 channels (Hydrocel Geodesic Sensor Net, NetAmps 300 amplifier, NetStation 4.2. software—Electrical Geodesics Inc.). The electrodes were referenced to the vertex (Cz) during recording and re-referenced offline to the average reference. Impedances were kept below 50 kΩ for all electrodes (high input impedance system). Data was pre-processed using EEGLAB (v11.4.2.2b) (36, 37) and custom MATLAB scripts. Continuous EEG records were downsampled to 250 Hz and band-pass filtered (0.2–30 Hz) and submitted to an Independent Components Analysis (ICA) decomposition after interpolation of bad channels (maximum of 10% per record). Correction of eye blink artifacts was carried out semi-automatically using the CORRMAP EEGLAB plug-in (38). We used a correlation threshold of 0.80 to identify the artifactual ICs and then subtracted their activity from the EEG data. Pre- and post-subtraction EEG traces were visually inspected to ensure that signals were not altered outside the time windows of eye blinks. EEG records were segmented into 1,000 ms epochs (−200 to 800 ms in peri-stimulus time). Epochs with voltages below −100 or above 100 μV were automatically rejected and all segments were subjected to visual inspection and manual artifact rejection. Finally, all epochs were baseline corrected (200 ms pre-stimulus) and averaged by condition for each participant.

### ERP Analysis

Grand-averaged ERPs were computed and visually inspected to ensure that the expected ERP morphology was present. We analyzed frontal scalp regions in the left (i.e., FC5 cluster: E24, E27, E28, E29, E34, and E35) and right hemispheres (i.e., FC6 cluster: E110, E111, E116, E123, E124, and E117), where the frontotemporal positivity to voices has been shown to be maximal (5). Time-windows for the N1 and P2 components were based on the visual inspection of the grand-averages and on the typical settings described in the literature on auditory ERPs. Therefore, peak amplitudes were quantified as the minimum voltage in the 80–200 ms post-stimulus presentation time-window for N1 and as the maximum voltage in the 200–300 ms time-window for P2. Peaks were extracted for each participant at each channel and then averaged per train position (i.e., 1–7, for bird songs and voices) and stimulus type (i.e., bird songs, vocalization, and pure tone).

### Statistical Analysis

For the analysis of behavioral data on the target detection task, responses were divided according to the sound (i.e., bird songs or voices) that was presented previous to the target (i.e., pure tone) and submitted to paired-sample *t*-tests to assess possible differences in reaction times (RTs). Additionally, independent samples *t*-tests were performed to assess group differences (i.e., patients vs. controls) in RTs (ms) and accuracy (percentage of times the target tone was correctly identified).

For neural data, separate Mixed Repeated Measure ANOVAs were conducted for N1 and P2. Pure tones (H1) were analyzed by hemisphere (within-participants factor: left, right) and group (between-participants factor: controls, patients). To replicate the conditions from previous studies that did not analyze the seven positions in the train (5), we explored group differences in the processing of complex stimulus (H2) only for the first stimulus of the train (within-participants factors: hemisphere—left, right, and stimulus category—bird songs, human voices). Finally, to explore the habituation properties of these ERPs for bird and human voices stimuli, we measured the decrease in amplitude across stimulus repetition in each train from S1 to S7 using linear contrasts (H3). As such this model entered with between groups and within-participants factors (train sequence—stimuli repetition from 1 to 7; stimulus category—voices, bird songs; hemisphere—left, right). All the pairwise comparisons were corrected for multiple comparisons using the Bonferroni Correction, and effect sizes were calculated (i.e., partial eta squared and Cohen's *d*).

## Results

### Behavioral Results: Target/ Pure-Tone Detection

RTs were significantly longer for targets following the presentation of human voices (*M* = 423.70, *SD* = 93.78) compared to targets following bird songs (*M* = 392.60, *SD* = 80.15), *t*_(50)_ = −5.26, *p* < 0.001, *d* = 0.737. For targets following the presentation of bird songs, patients had significantly longer RTs (*M* = 419.14, *SD* = 80.85) compared to controls (*M* = 364.98, *SD* = 70.2), *t*_(49)_ = 2.54, *p* = 0.014, *d* = 0.713. Patients also had significantly longer RTs for targets following human voices (*M* = 461.95, *SD* = 92.71) compared to controls (*M* = 383.92, *SD* = 78.32), *t*_(49)_ = 3.24, *p* = 0.002, *d* = 0.909.

The differences in accuracy between patients (*M* = 96.50%, *SD* = 7.01) and controls (*M* = 99.19%, *SD* = 2.19) were not statistically different, *t*_(51)_ = −1.87, *p* = 0.067, *d* = 0.518.

### H1: Category— Simple Sound (Pure Tones—Target Stimuli)

**Figure 2:**
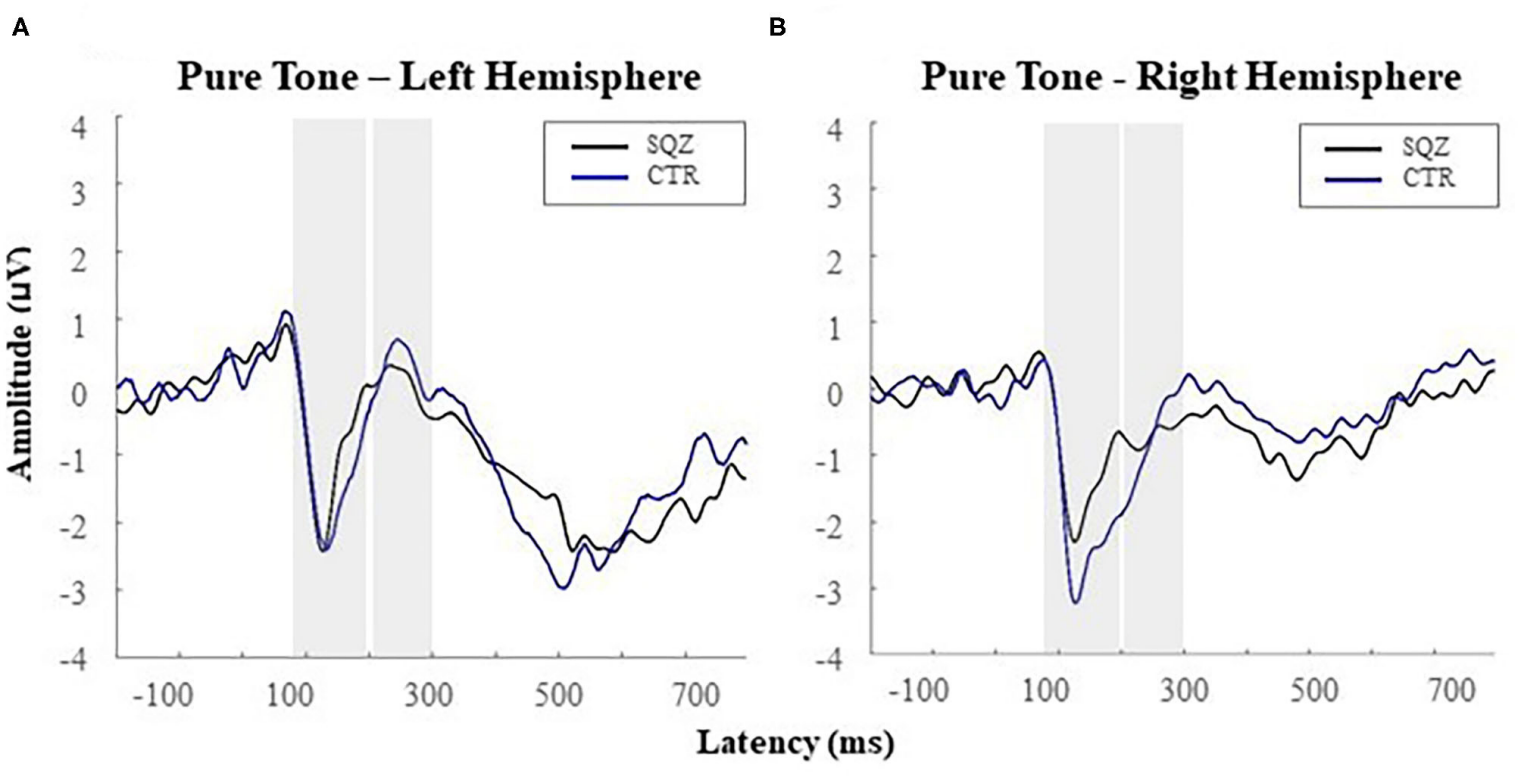
Auditory event-related potentials [i.e., N1 (80–200 ms); P2 (200–300 ms)] for the pure tone (i.e., the target tone) in patients with schizophrenia (SQZ) and healthy controls (CTR): **(A)** in the left hemisphere (i.e., cluster FC5) and **(B)** in the right hemisphere (i.e., cluster FC6).

At the N1 time-window, a significant hemisphere^*^group interaction was found, *F*_(1, 51)_ = 4.52, *p* = 0.038, *η*^2^_*p*_ =0.081, revealing lower N1 amplitudes in patients (*M* = −3.02, *SD* = 1.48) compared to controls (*M* = −4.42, *SD* = 1.87) in the right hemisphere, *t*_(51)_ = −3.01, *p* = 0.024, *d* = 0.83 (see Figure 2). No other significant effects were found for the N1.

For P2, a significant effect was found for hemisphere, *F*_(1, 51)_ = 5.35, *p* = 0.025, *η*^2^_*p*_ = 0.095, indicating higher amplitudes for the left (*M* = 1.82, *SD* = 1.89) comparing to the right hemisphere (*M* = 1.02, *SD* = 2.12), *t*_(52)_ = 2.34, *p* = 0.023, *d* = 0.31, for both groups. No other significant effects were found for the P2.

All descriptive statistics of peak amplitudes and results of the separate Mixed Repeated Measure ANOVAs for N1 and P2 can be found in the Supplementary Tables 1, 2).

### H2: Category—Complex Sounds (Voices vs. Bird Songs)

There were no main effects of stimulus category, nor category^*^group or category^*^group^*^hemisphere interactions on N1 amplitude (see also Supplementary Table 3).

**Figure 3:**
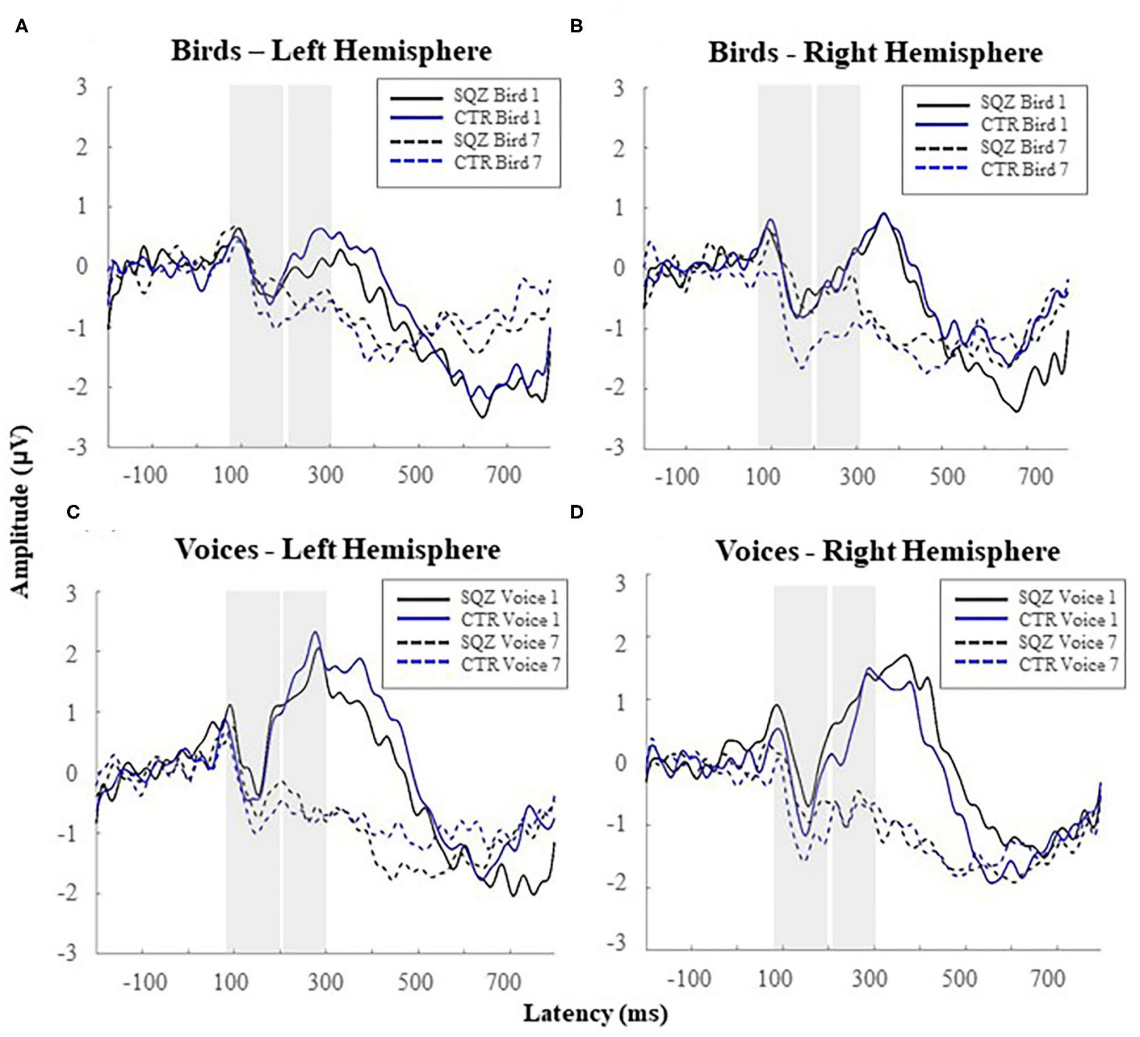
Auditory event-related potentials [i.e., N1 (80–200 ms); P2 (200–300 ms)] for complex sounds in patients with schizophrenia (SQZ) and healthy controls (CTR): **(A)** for bird songs in the left hemisphere (i.e., cluster FC5) and **(B)** right hemisphere (i.e., cluster FC6); and **(C)** for human voices in the left hemisphere (i.e., cluster FC5) and **(D)** the right hemisphere (i.e., cluster FC6).

A main effect of category was found on P2 amplitude, *F*_(1, 51)_ = 78.63, *p* < 0.001, *η*^2^_*p*_ = 0.607, indicating that voices elicited increased P2 amplitude (see Figure 3) than bird songs for both groups (all *p* < 0.036). No group or hemisphere^*^group interactions were found (see also Supplementary Table 4).

### H3: Habituation Effects

For N1, we found a significant linear contrast effect of order for human voices, *F*_(1, 51)_ = 10.00, *p* = 0.003, *η*^2^_*p*_ =0.164, but not for bird songs *F*_(1, 51)_ = 1.75, *p* = 0.192, *η*^2^_*p*_ = 0.033. No further effects were observed.

**Figure 4:**
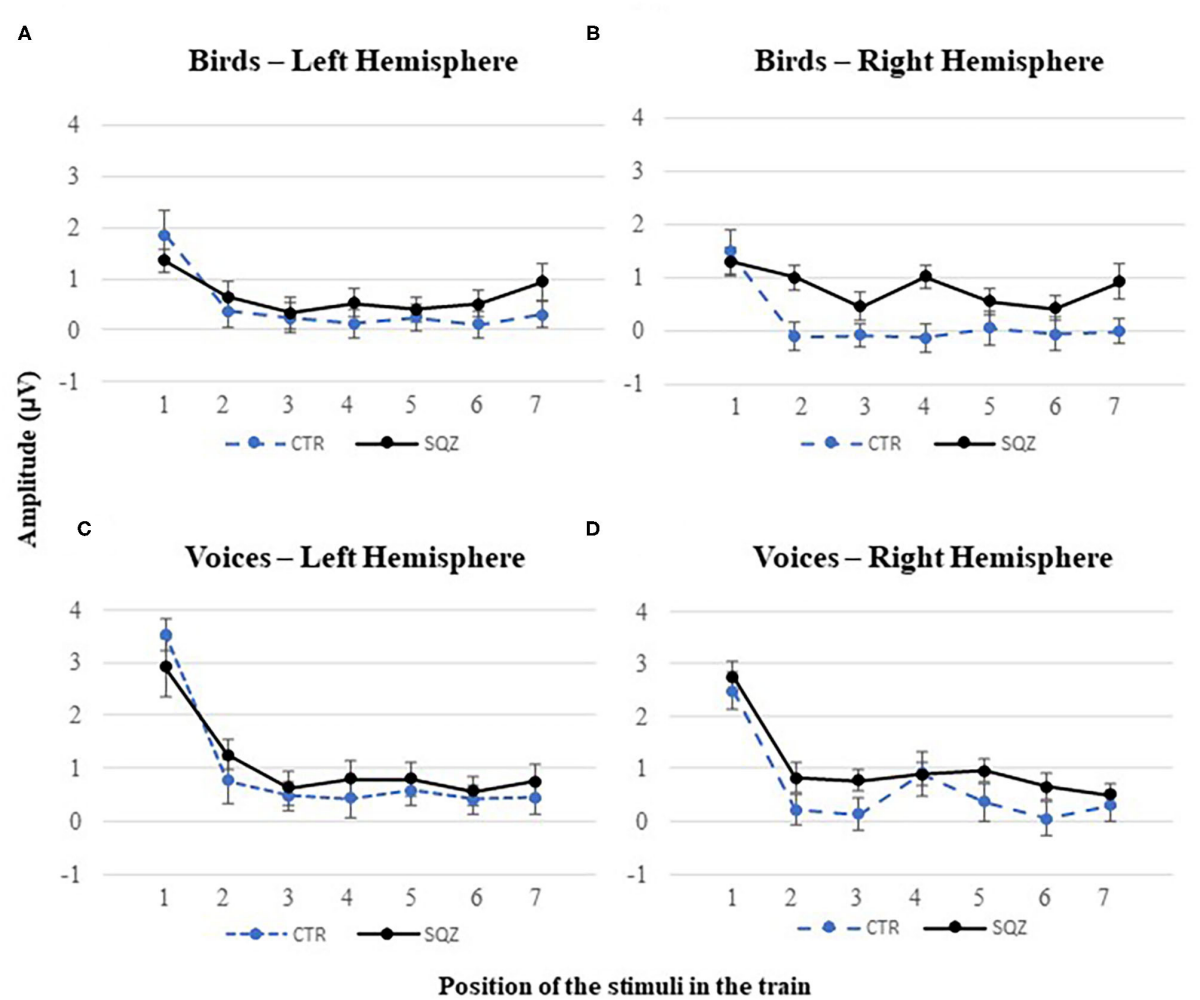
P2 habituation effect in patients with schizophrenia (SQZ) and healthy controls (CTR) for complex sounds. P2 peak amplitudes to each position of the stimuli in the train for: **(A)** bird songs in the left hemisphere (i.e., cluster FC5) and **(B)** the right hemisphere (i.e., cluster FC6); and **(C)** human voices in the left hemisphere (i.e., cluster FC5) and **(D)** the right hemisphere (i.e., cluster FC6), for each position in the train.

For P2, we found a significant linear contrast effect of order in bird songs *F*_(1, 51)_ = 21.71, *p* < 0.001, *η*^2^_*p*_ = 0.299, and voices, *F*_(1, 51)_ = 81.10, *p* < 0.001, *η*^2^_*p*_ = 0.614. Also, a significant interaction order^*^group was found in P2 for bird songs, *F*_(1, 51)_ = 4.21, *p* < 0.045, *η*^2^_*p*_ = 0.760 [vs. human voices, *F*_(1, 51)_ = 0.294, *p* = 0.590, *η*^2^_*p*_ = 0.006], independently of the hemisphere, *F*_(1, 51)_ = 1.02, *p* = 0.319, *η*^2^_*p*_ = 0.020. This finding (see Figures 3, 4) represents a gradual decrease in P2 amplitude for controls from Bird1 to Bird7 at both hemispheres (Bird1: *M*_*FC*5_ = 1.85, *SD* = 2.44, *M*_*FC*6_ = 1.49, *SD* = 2.21; Bird7: *M*_*FC*5_ = 0.30, *SD* = 1.38, *M*_*FC*6_ = −0.01, *SD* = 1.31, both *p*s < 0.040). This habituation effect on birds was not observed in patients (Bird1: *M*_*FC*5_ = 1.35, *SD* = 1.19, *M*_*FC*6_ = 1.31, *SD* = 1.41; Bird7: *M*_*FC*5_ = 0.94, *SD* = 0.86, *M*_*FC*6_ = −0.92, *SD* = 1.75, all *p* > 0.820).

## Discussion

The habituation of the auditory N1 and P2 components of the ERPs remains largely unexplored in schizophrenia, especially when compared with the body of research directed to P50 and P300 components. The current work intended to expand the knowledge on these components. For this purpose, N1 and P2 amplitudes were measured in a roving paradigm. Our hypotheses considered not only the habituation properties of N1 and P2, but also the different modulations induced by distinct stimulus categories. Supporting H1, we found a diminished N1 amplitude for pure tones in patients at the right frontotemporal region. In turn, H2 was not confirmed since human voices elicited higher P2 amplitudes than bird song at frontotemporal locations for both groups. Finally, H3 was partially verified, since habituation effects were moderated by group differences in P2, but not in N1 and only for bird songs. Our results showed that human voices (but not bird songs) specifically elicited a pattern of habituation in N1 in both groups. However, P2 amplitudes faded with train repetitions of bird songs only in the control group, while habituation effects to human voices were present in both groups.

The results concerning H1-Pure tones—resemble previous findings from oddball tasks, since patients with schizophrenia seem to have difficulties in allocating attention to target/relevant auditory stimuli, as reflected in the attenuation of the N1 component (9). However, for H2 we found no evidence that deficits in voice processing in schizophrenia would lead to a diminished P2. Notwithstanding, our results support previous literature that reported higher P2 amplitudes elicited by human voices compared to bird songs at Frontocentral locations (5, 7, 11), but these results did not interact with group. In other words, patients showed similar response amplitudes as controls in the initial discrimination of human voices, despite the literature describing abnormalities in P2 modulation for this group (24, 25). It may be the case that the P2 reflects an early preference for human voice processing that goes in line with the social nature of the human brain and the priority given to social cues within the species (6, 7). Although there have been descriptions of abnormalities regarding voice processing when negative emotional content is added (29), no differences have been found for non-semantic speech and neutral voice sounds (28, 30), suggesting that early stages of voice processing might be unaffected in early schizophrenia.

Taking together H1 and H2, N1 seems to mirror an orienting response toward target/simple stimuli that is impaired in schizophrenia, while P2 is likely to index more specific, elaborated categorization processes of complex sounds that are not necessarily impaired in schizophrenia. Given the differences in functional significance, these findings further unveil that the current experimental manipulation led to the dissociation of N1 and P2 amplitude, which is also reflected in habituation processes.

Furthermore, our results provide evidence that both N1 and P2 are habituating components (1, 13), notwithstanding that habituation effects are category dependent for N1. Interestingly, habituation effects were moderated by group differences in P2, but not in N1. This partially contradicts H3, as we expected a general dysfunction in habituation processes at both time-windows and stimuli in patients (25). Importantly, this effect was specific to birds and not voices, with patients showing the expected pattern of non-habituation for birds in P2. Patterns of abnormal habituation have been found for this component in schizophrenia using simple stimuli (25), and the same seems to be the case for bird songs [complex non-vocal stimuli. The specificity regarding auditory processing of the human voice may help to understand this differential effect. The early positivity that occurs around the P2 time-window has been described as sensitive to voices with increased amplitudes for these stimuli when compared with other sound categories (5). Our results corroborate these findings i.e., human voices elicited higher P2 amplitudes than bird songs for both groups]. In fact, the preference for sensory information of conspecifics is observable on the visual N170 and its increased amplitudes for faces when compared with objects (39, 40). Thus, it is possible that both auditory and visual preference for conspecific stimuli plays a relevant role in social information processing (41). From the available evidence on this matter, one study by Williams et al. (42) analyzed the habituation of brain activations in response to faces in schizophrenia. No habituation was found in several brain regions of patients (e.g., primary visual cortex and hippocampus), but when looking at the putative generator of the N170—the Fusiform Face Area (43)—patients and controls showed a similar pattern of habituation to faces. It remains plausible therefore that conspecific signals (such as voice and faces) are processed by specialized brain modules that are somewhat independent of other stimulus categories, considering how crucial it is for communication and social interactions. Developmental studies highlight indeed that preference for voices emerges early, with habituation to human voices (vs. environmental stimuli) being observable in preschoolers (44). Additionally, brain regions responsible for voice processing are thought to maturate during the 1st year of life (45), whereas P2 modulations by stimulus category and habituation are mature in 5-year-old toddlers (46, 47). So, it is possible that voice processing specializes much earlier than the onset of schizophrenia symptoms and possibly explaining how first-episode patients exhibit normal patterns of brain response to neutral non-speech human voices.

Limitations of this study comprise the absence of a formal assessment of mental and hearing abilities and the inclusion of groups on antipsychotic medication, making it impossible to dissociate the effects of the medication from the abnormalities found, as it has been previously suggested [e.g., (48)]. Also, only mid-latency ERP components were studied, not allowing us to investigate later stages of the auditory processing. Future research should consider additional stages of voice processing and investigate more nuanced associations with schizophrenia symptoms, such as hallucinations, as possible predictive factors of impairment [see e.g., (33)]. Nonetheless, regarding abnormal habituation for bird songs, it is important that future research uses other stimuli to understand if this pattern of habituation is generalized for non-voices or specific for bird songs. In addition, our results show that preference for human voices seems to be unaffected in early diagnosed schizophrenia patients. The investigation of abnormal patterns of brain activity in patients with schizophrenia, such as the ones found in the current work, allows researchers to better understand the impairments and symptoms of this pathology. More specifically, the ability to filter out irrelevant or repetitive information in early and later stages of information processing. This growing knowledge may be crucial for the future development of neurobiologically-informed early-diagnosis, assessment, and treatment for schizophrenia.

## Data Availability Statement

The data that support the findings of this study are available from the corresponding author, Prune Mazer, upon reasonable request.

## Ethics Statement

The studies involving human participants were reviewed and approved by Local Ethics committee of the Faculty of Psychology and Educational Sciences of the University of Porto. The patients/participants provided their written informed consent to participate in this study.

## Author Contributions

FF-S, PA, TP, FB, and JM-T conceptualized the study: CC-R, TP, and CS collected the data: PM, IM, RP, FF-S, and TP analyzed the data: PM, IM, RP, and TP drafted the manuscript. All authors revised the manuscript.

## Conflict of Interest

The authors declare that the research was conducted in the absence of any commercial or financial relationships that could be construed as a potential conflict of interest.
