# Supplementary material for: Abnormal Habituation of the Auditory Event-Related Potential P2 Component in Patients With Schizophrenia
Source: Front Psychiatry. 2021 Mar 18;12:630406. doi: 10.3389/fpsyt.2021.630406 (PMC8012906; doi:10.3389/fpsyt.2021.630406)
Supplement: Supplementary file 1 [file Data_Sheet_1.docx]

***Supplementary Material***

1. **Validation study of the auditory stimuli**

Stimuli selection for the experimental task was based on a preliminary validation study of the different stimuli categories.

**1.1. Participants**

Ten healthy right-handed participants (8 females) took part in the validation study (mean age = 23.30, SD = 3.10, range [19, 30]). All participants reported being free from neurologic or psychiatric illness, having no history of head injuries or substance abuse, and having normal hearing. Informed consent was obtained from all participants before the beginning of the experiment.

**1.2. Stimuli and procedure**

Stimuli consisted of 84 sound samples: 35 bird songs that were selected from the “Chants d’oiseaux du Québec et de l’Amérique du Nord” (Peterson, 1990) audio CD; 6 pure tones with two frequencies (500Hz and 1000Hz) and three intensities (70, 80, and 90 dB SPL) that were created using Matlab (The MathWorks Inc., Natick, MA, USA); and 43 non-speech vocal sounds retrieved from http://vnl.psy.gla.ac.uk/resources/speed_of_voice_sounds. The sounds were edited according to the specifications from Charest and colleagues (2009): sampling rate of 22050 Hz, 16-bit resolution, duration of 200 ms (10 ms of rise and fall times) and root mean square (RMS) intensity normalization of the sounds.

All sounds were binaurally presented using E-Prime 2.0 software (Psychology Software Tools, Inc., Sharpsburg, PA, USA). The protocol consisted in the random presentation of each stimulus with 2.3 seconds of inter-stimulus-interval (ISI). Each sound was presented 5 times during the protocol. Participants were asked to classify each sound within one of four categories (i.e., pure tone, bird song, voices, or other).

**1.3. Statistical analyses and final stimuli set**

For each sound, both accuracy and mean reaction times were computed. The criteria for choosing the final stimuli set for the experimental task of the study was accuracy, defined as the percentage of correct identification of stimulus class (all equal or above 98% in the selected stimuli); and, in case of equal values of accuracy between sounds, the ones with faster mean reaction times were better ranked.

The final stimuli set consisted of 8 bird songs, 8 vocalizations, and 1 pure tone to serve as a target in the final experimental task.

|  | **Patients** (n=26) | | | | **Controls** (n=27) | | | |
| --- | --- | --- | --- | --- | --- | --- | --- | --- |
|  | **N1** | | **P2** | | **N1** | | **P2** | |
|  | **M** | **SD** | **M** | **SD** | **M** | **SD** | **M** | **SD** |
| **Left Hemisphere (FC5 cluster)** | | | | | | | | |
| Bird 1 | -1.66 | 1.16 | 1.35 | 1.19 | -1.53 | 1.99 | 1.85 | 2.44 |
| Bird 2 | -1.81 | 1.49 | 0.65 | 1.46 | -2.03 | 1.30 | 0.37 | 1.75 |
| Bird 3 | -2.00 | 1.46 | 0.34 | 1.62 | -2.04 | 1.51 | 0.24 | 1.51 |
| Bird 4 | -1.94 | 1.30 | 0.53 | 1.48 | -2.08 | 1.46 | 0.13 | 1.48 |
| Bird 5 | -2.10 | 1.02 | 0.40 | 1.25 | -1.91 | 1.71 | 0.25 | 1.41 |
| Bird 6 | -1.77 | 1.08 | 0.51 | 1.32 | -1.82 | 1.58 | 0.11 | 1.36 |
| Bird 7 | -1.43 | 1.14 | 0.94 | 1.86 | -1.87 | 1.72 | 0.30 | 1.38 |
| Voc 1 | -1.18 | 1.38 | 2.91 | 1.64 | -1.55 | 1.33 | 3.53 | 2.92 |
| Voc 2 | -1.31 | 1.45 | 1.24 | 2.16 | -1.55 | 1.52 | 0.76 | 1.51 |
| Voc 3 | -1.89 | 1.22 | 0.63 | 1.39 | -2.11 | 1.86 | 0.47 | 1.67 |
| Voc 4 | -1.76 | 1.22 | 0.79 | 1.81 | -2.09 | 1.37 | 0.43 | 1.85 |
| Voc 5 | -1.87 | 1.20 | 0.79 | 1.35 | -1.72 | 1.64 | 0.57 | 1.69 |
| Voc 6 | -1.88 | 1.14 | 0.56 | 1.33 | -1.91 | 1.26 | 0.41 | 1.41 |
| Voc 7 | -1.62 | 1.49 | 0.75 | 1.58 | -1.83 | 1.66 | 0.45 | 1.79 |
| Pure tones | -3.06 | 1.72 | 1.56 | 1.30 | -3.42 | 2.68 | 2.07 | 2.32 |
|  |  |  |  |  |  |  |  |  |
| **Right Hemisphere (FC6 cluster)** | | | | | | | | |
| Bird 1 | -1.62 | 1.25 | 1.31 | 1.40 | -1.92 | 1.74 | 1.49 | 2.21 |
| Bird 2 | -1.25 | 0.92 | 1.00 | 1.16 | -2.08 | 1.62 | -0.12 | 1.42 |
| Bird 3 | -2.03 | 1.25 | 0.45 | 1.39 | -2.17 | 1.19 | -0.08 | 1.14 |
| Bird 4 | -1.41 | 1.03 | 1.02 | 1.08 | -2.35 | 1.30 | -0.13 | 1.37 |
| Bird 5 | -1.87 | 1.36 | 0.55 | 1.27 | -2.27 | 1.48 | 0.05 | 1.72 |
| Bird 6 | -1.76 | 1.06 | 0.43 | 1.25 | -2.25 | 1.74 | -0.06 | 1.63 |
| Bird 7 | -1.70 | 1.25 | 0.92 | 1.75 | -2.40 | 1.46 | -0.01 | 1.31 |
| Voc 1 | -1.49 | 1.12 | 2.76 | 1.42 | -2.10 | 1.51 | 2.48 | 1.88 |
| Voc 2 | -1.70 | 1.96 | 0.82 | 1.48 | -2.13 | 1.54 | 0.22 | 1.50 |
| Voc 3 | -1.83 | 1.14 | 0.77 | 1.07 | -2.42 | 1.61 | 0.13 | 1.56 |
| Voc 4 | -2.05 | 1.19 | 0.89 | 1.18 | -2.46 | 1.56 | 0.91 | 2.27 |
| Voc 5 | -1.52 | 0.83 | 0.96 | 1.19 | -2.25 | 1.59 | 0.36 | 1.84 |
| Voc 6 | -1.88 | 1.15 | 0.66 | 1.25 | -2.41 | 1.52 | 0.05 | 1.71 |
| Voc 7 | -1.89 | 1.36 | 0.49 | 1.17 | -2.49 | 1.42 | 0.29 | 1.51 |
| Pure tones | -3.02 | 1.48 | 0.84 | 1.52 | -4.42 | 1.87 | 1.19 | 2.59 |
|  |  |  |  |  |  |  |  |  |

Supplementary Table S1. Descriptive statistics for N1 and P2 amplitudes.

|  | **df_c_, df_e_** | **Mean square** | ***F*** | **Sig.** | **η^2^_p_** |
| --- | --- | --- | --- | --- | --- |
| **N1** |  |  |  |  |  |
| Hemisphere | 1, 51 | 6.168 | 3.902 | .054 | .071 |
| Hemisphere*Group | 1, 51 | 7.138 | 4.516 | .038 | .081 |
| **P2** |  |  |  |  |  |
| Hemisphere | 1, 51 | 17.088 | 5.348 | .025 | .095 |
| Hemisphere*Group | 1, 51 | 0.148 | 0.046 | .830 | .001 |

Supplementary Table S2. Separate Mixed Repeated Measure ANOVAs for N1 and P2 for pure tones.

|  | **df_c_, df_e_** | **Mean square** | ***F*** | **Sig.** | **η^2^_p_** |
| --- | --- | --- | --- | --- | --- |
| **N1** |  |  |  |  |  |
| Category | 1, 51 | 0.573 | 0.552 | .461 | .011 |
| Category*Group | 1, 51 | 2.221 | 2.138 | .150 | .040 |
| Hemisphere | 1, 51 | 4.797 | 2.297 | .136 | .043 |
| Hemisphere*Group | 1, 51 | 1.523 | 0.729 | .397 | .014 |
| Category*Hemisphere | 1, 51 | 0.853 | 0.79 | .378 | .015 |
| Category*Hemisphere*Group | 1, 51 | 0.137 | 0.127 | .723 | .002 |

Supplementary Table S3. Separate Mixed Repeated Measure ANOVAs for N1 for complex sounds (voices vs birds).

|  | **df_c_, df_e_** | **Mean square** | ***F*** | **Sig.** | **η^2^_p_** |
| --- | --- | --- | --- | --- | --- |
| **P2** |  |  |  |  |  |
| Category | 1, 51 | 106.387 | 78.629 | < .001 | .607 |
| Category*Group | 1, 51 | 0.434 | 2.138 | .574 | .006 |
| Hemisphere | 1, 51 | 8.563 | 2.297 | .111 | .049 |
| Hemisphere*Group | 1, 51 | 4.830 | 0.729 | .229 | .028 |
| Category*Hemisphere | 1, 51 | 2.129 | 0.79 | .225 | .029 |
| Category*Hemispher*Group | 1, 51 | 1.113 | 0.127 | .378 | .015 |

Supplementary Table S4. Separate Mixed Repeated Measure ANOVAs for P2 for complex sounds (voices vs birds).

**References**

Charest, I., Pernet, C. R., Rousselet, G. A., Quiñones, I., Latinus, M., Fillion-Bilodeau, S., Chartrand, J.-P., & Belin, P. (2009). Electrophysiological evidence for an early processing of human voices. *BMC Neuroscience, 10*, 127. doi:10.1186/1471-2202/10/127

Peterson, R. T. (1990). *Chants d'oiseaux du Québec e de l'Est de l'Amerique du Nord* [CD]. Ottawa: Cornell Laboratory of Ornithology and Interactive Audio.
